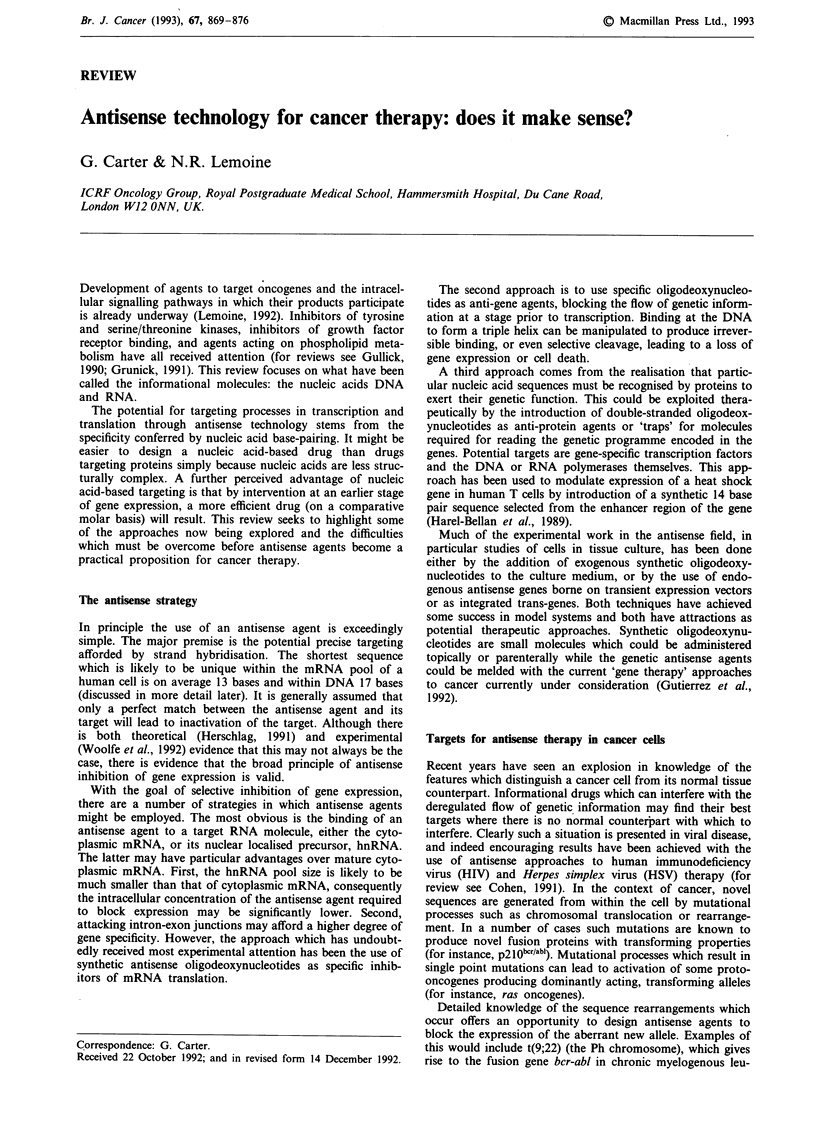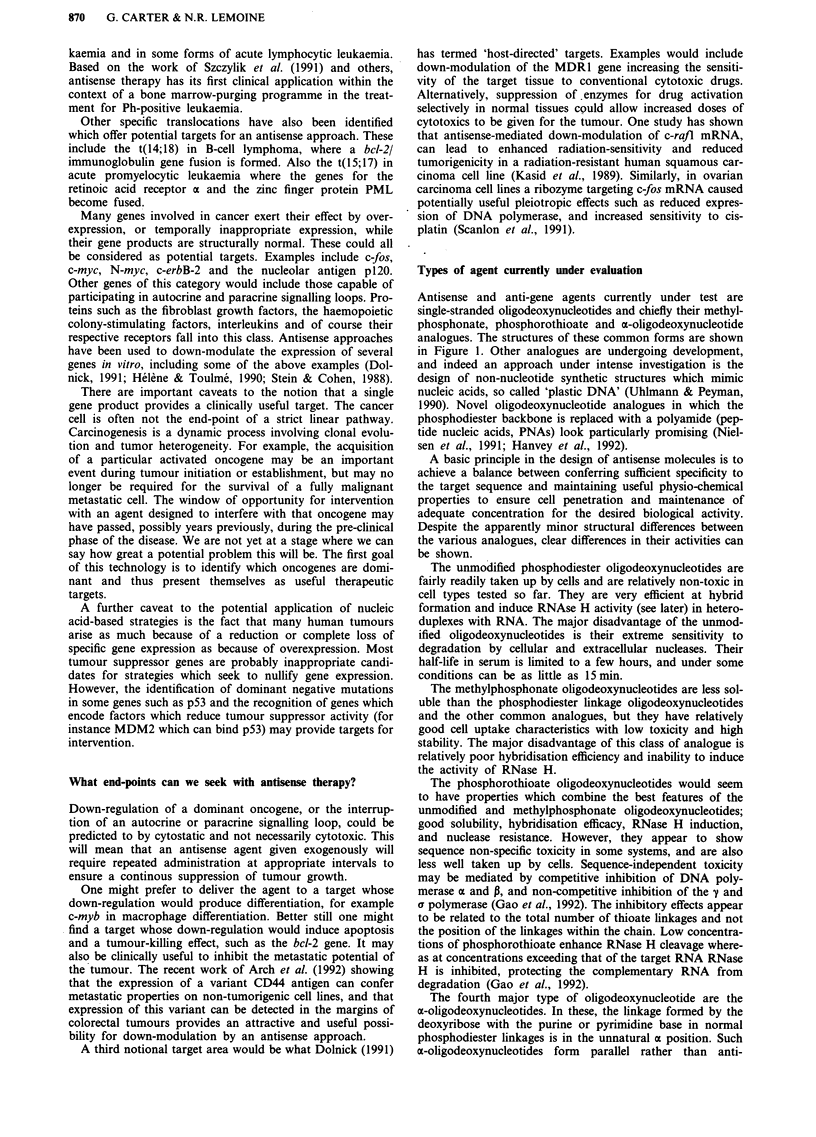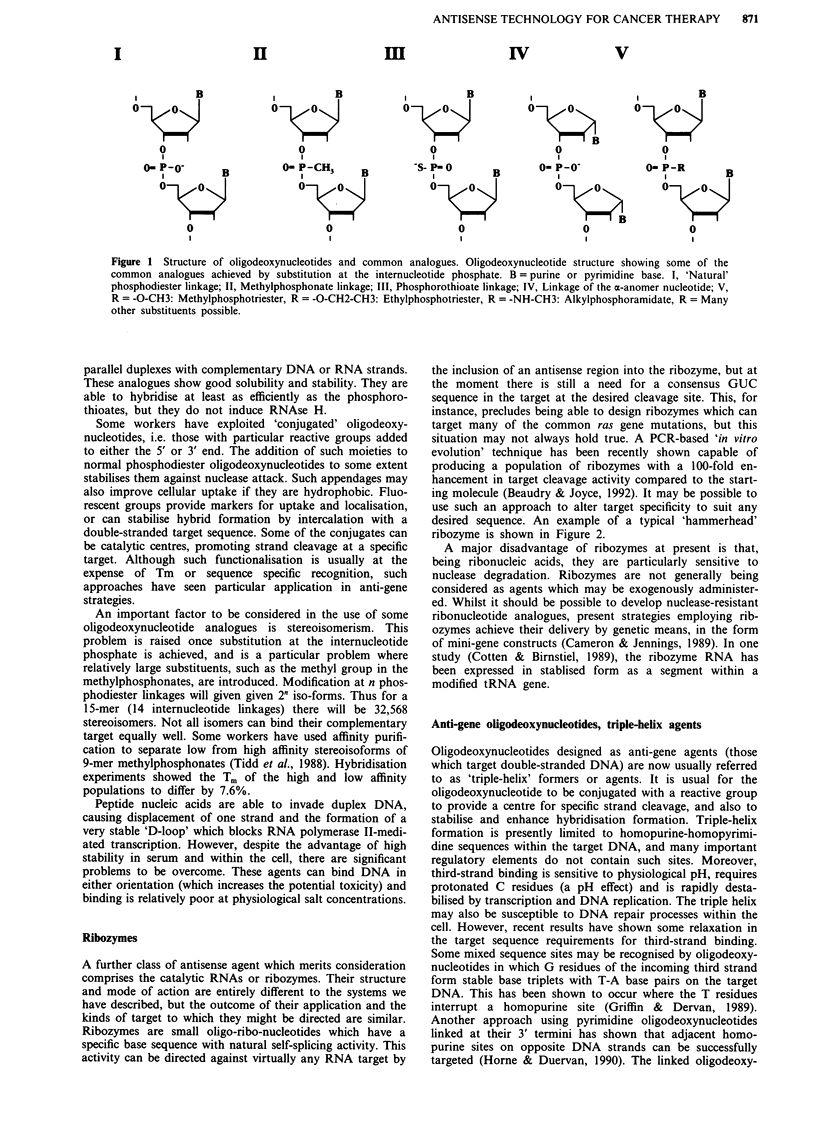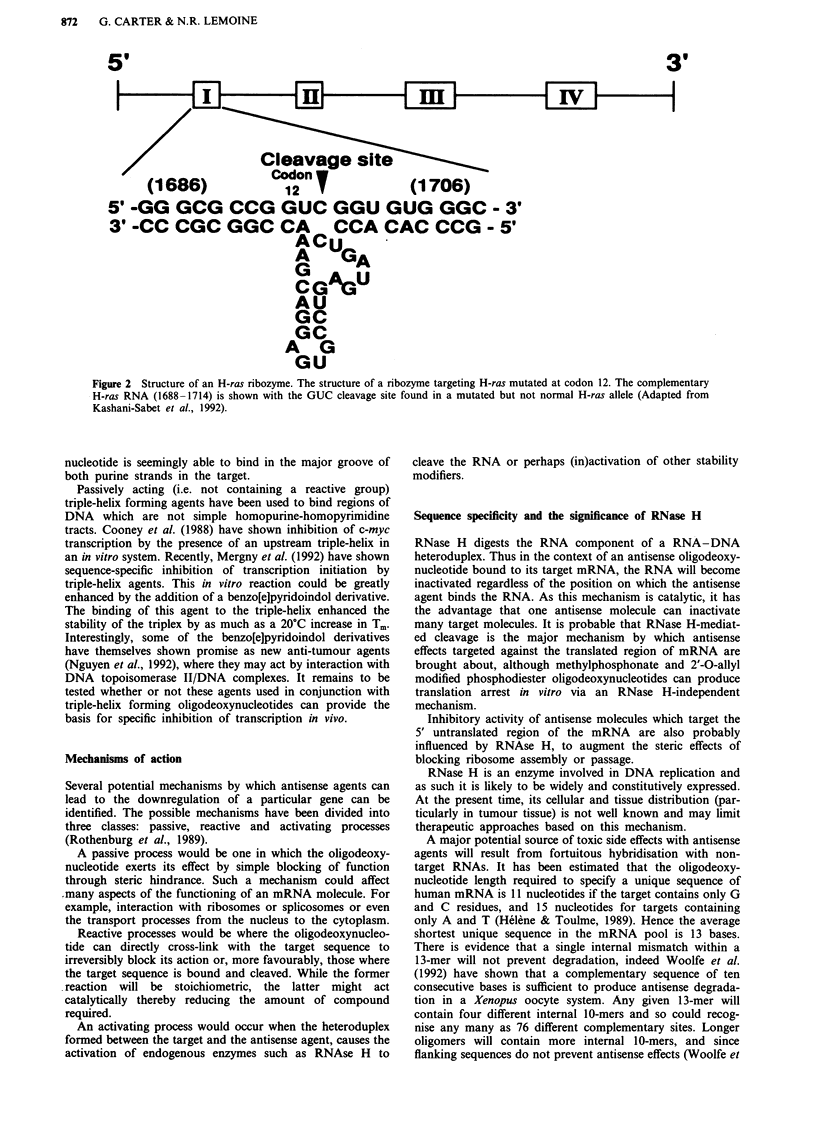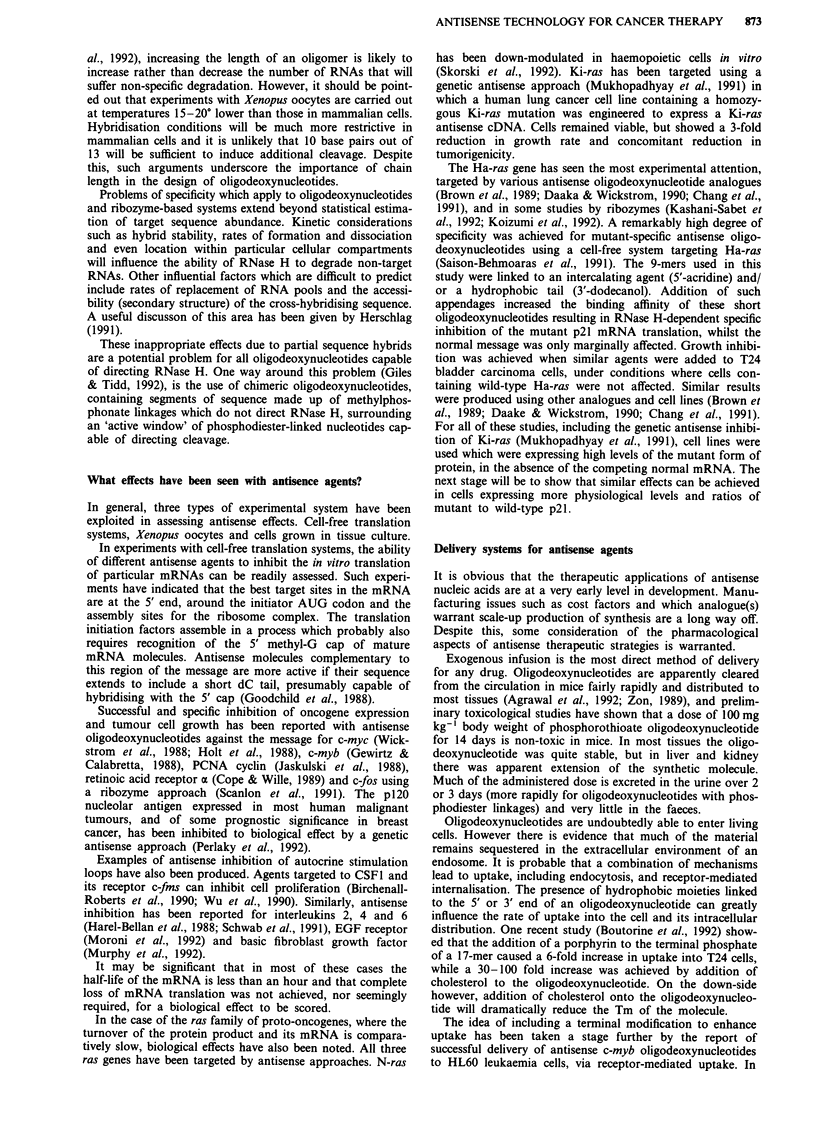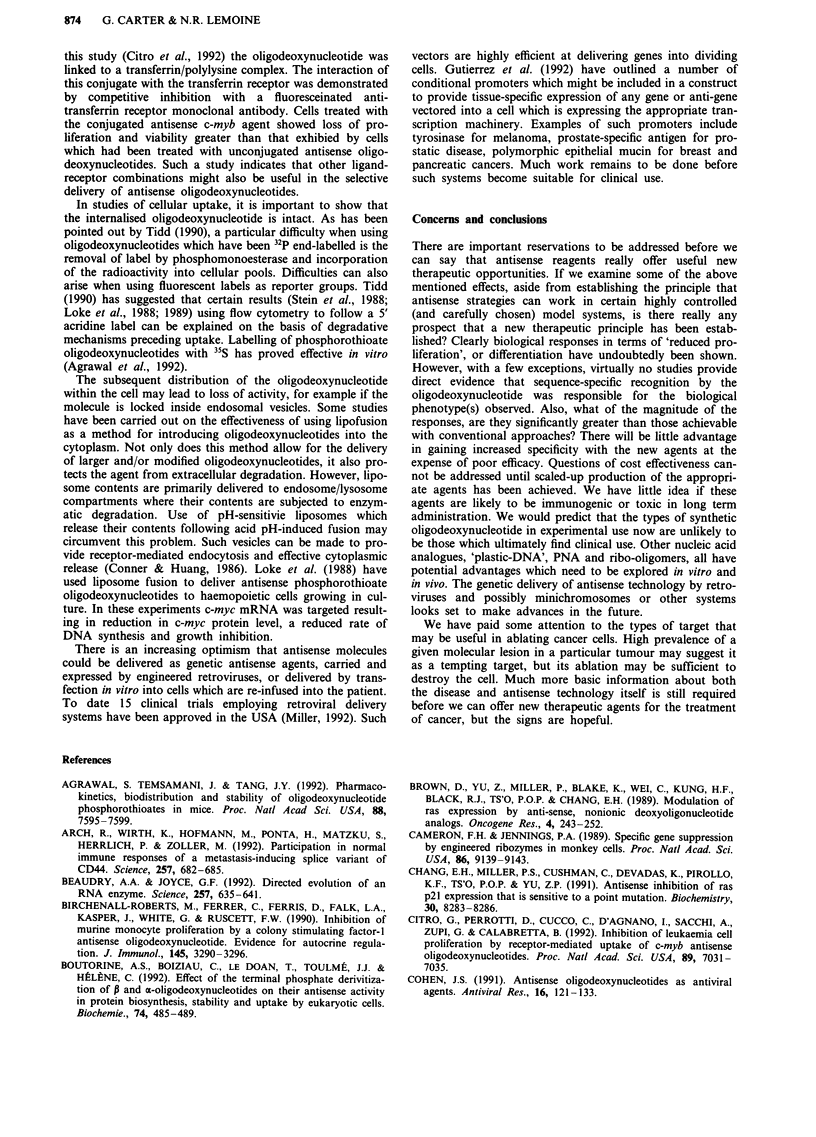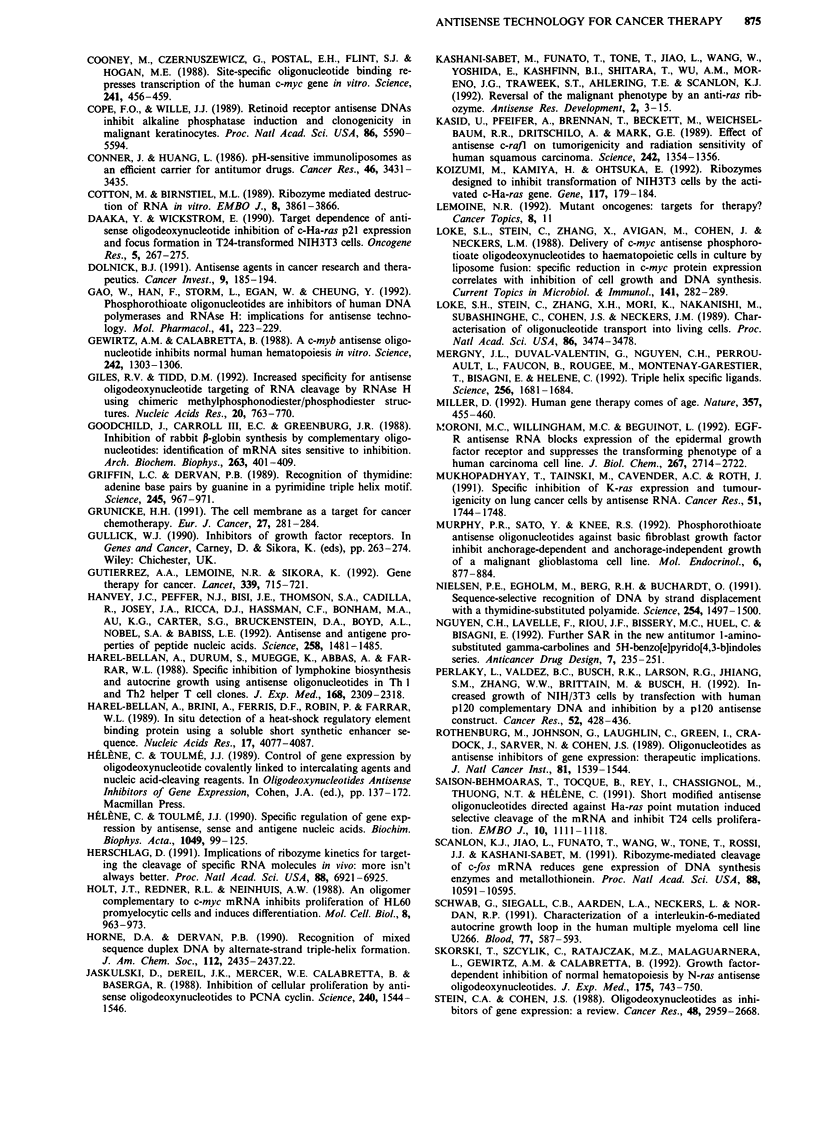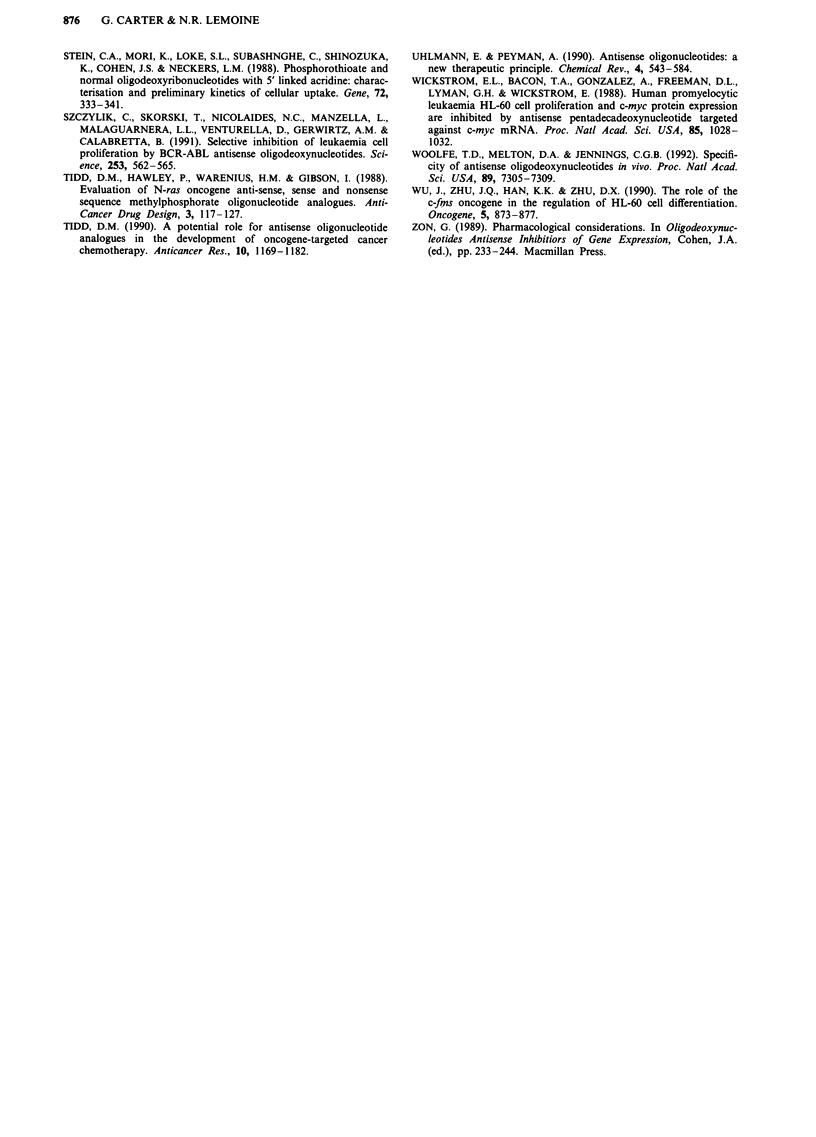# Antisense technology for cancer therapy: does it make sense?

**DOI:** 10.1038/bjc.1993.164

**Published:** 1993-05

**Authors:** G. Carter, N. R. Lemoine

**Affiliations:** ICRF Oncology Group, Royal Postgraduate Medical School, Hammersmith Hospital, London, UK.


Br. J. Cancer (1993), 67, 869–876 © Macmillan Press Ltd., 1993

REVIEW

### Antisense technology for cancer therapy: does it make sense?

G. Carter & N.R. Lemoine

*ICRF Oncology Group, Royal Postgraduate Medical School, Hammersmith Hospital, Du Cane Road, London W12 0NN, UK.*

Development of agents to target oncogenes and the intracellular signalling pathways in which their products participate is already underway (Lemoine, 1992). Inhibitors of tyrosine and serine/threonine kinases, inhibitors of growth factor receptor binding, and agents acting on phospholipid metabolism have all received attention (for reviews see Gullick, 1990; Grunick, 1991). This review focuses on what have been called the informational molecules: the nucleic acids DNA and RNA.

The potential for targeting processes in transcription and translation through antisense technology stems from the specificity conferred by nucleic acid base-pairing. It might be easier to design a nucleic acid-based drug than drugs targeting proteins simply because nucleic acids are less structurally complex. A further perceived advantage of nucleic acid-based targeting is that by intervention at an earlier stage of gene expression, a more efficient drug (on a comparative molar basis) will result. This review seeks to highlight some of the approaches now being explored and the difficulties which must be overcome before antisense agents become a practical proposition for cancer therapy.

### The antisense strategy

In principle the use of an antisense agent is exceedingly simple. The major premise is the potential precise targeting afforded by strand hybridisation. The shortest sequence which is likely to be unique within the mRNA pool of a human cell is on average 13 bases and within DNA 17 bases (discussed in more detail later). It is generally assumed that only a perfect match between the antisense agent and its target will lead to inactivation of the target. Although there is both theoretical (Herschlag, 1991) and experimental (Woolfe *et al.*, 1992) evidence that this may not always be the case, there is evidence that the broad principle of antisense inhibition of gene expression is valid.

With the goal of selective inhibition of gene expression, there are a number of strategies in which antisense agents might be employed. The most obvious is the binding of an antisense agent to a target RNA molecule, either the cytoplasmic mRNA, or its nuclear localised precursor, hnRNA. The latter may have particular advantages over mature cytoplasmic mRNA. First, the hnRNA pool size is likely to be much smaller than that of cytoplasmic mRNA, consequently the intracellular concentration of the antisense agent required to block expression may be significantly lower. Second, attacking intron-exon junctions may afford a higher degree of gene specificity. However, the approach which has undoubtedly received most experimental attention has been the use of synthetic antisense oligodeoxynucleotides as specific inhibitors of mRNA translation.

Correspondence: G. Carter.
Received 22 October 1992; and in revised form 14 December 1992.

The second approach is to use specific oligodeoxynucleotides as anti-gene agents, blocking the flow of genetic information at a stage prior to transcription. Binding at the DNA to form a triple helix can be manipulated to produce irreversible binding, or even selective cleavage, leading to a loss of gene expression or cell death.

A third approach comes from the realisation that particular nucleic acid sequences must be recognised by proteins to exert their genetic function. This could be exploited therapeutically by the introduction of double-stranded oligodeoxynucleotides as anti-protein agents or 'traps' for molecules required for reading the genetic programme encoded in the genes. Potential targets are gene-specific transcription factors and the DNA or RNA polymerases themselves. This approach has been used to modulate expression of a heat shock gene in human T cells by introduction of a synthetic 14 base pair sequence selected from the enhancer region of the gene (Harel-Bellan *et al.*, 1989).

Much of the experimental work in the antisense field, in particular studies of cells in tissue culture, has been done either by the addition of exogenous synthetic oligodeoxynucleotides to the culture medium, or by the use of endogenous antisense genes borne on transient expression vectors or as integrated trans-genes. Both techniques have achieved some success in model systems and both have attractions as potential therapeutic approaches. Synthetic oligodeoxynucleotides are small molecules which could be administered topically or parenterally while the genetic antisense agents could be melded with the current 'gene therapy' approaches to cancer currently under consideration (Gutierrez *et al.*, 1992).

### Targets for antisense therapy in cancer cells

Recent years have seen an explosion in knowledge of the features which distinguish a cancer cell from its normal tissue counterpart. Informational drugs which can interfere with the deregulated flow of genetic information may find their best targets where there is no normal counterpart with which to interfere. Clearly such a situation is presented in viral disease, and indeed encouraging results have been achieved with the use of antisense approaches to human immunodeficiency virus (HIV) and *Herpes simplex* virus (HSV) therapy (for review see Cohen, 1991). In the context of cancer, novel sequences are generated from within the cell by mutational processes such as chromosomal translocation or rearrangement. In a number of cases such mutations are known to produce novel fusion proteins with transforming properties (for instance, $p210^{bcr/abl}$). Mutational processes which result in single point mutations can lead to activation of some proto-oncogenes producing dominantly acting, transforming alleles (for instance, *ras* oncogenes).

Detailed knowledge of the sequence rearrangements which occur offers an opportunity to design antisense agents to block the expression of the aberrant new allele. Examples of this would include t(9;22) (the Ph chromosome), which gives rise to the fusion gene *bcr-abl* in chronic myelogenous leukaemia and in some forms of acute lymphocytic leukaemia. Based on the work of Szczylik *et al.* (1991) and others, antisense therapy has its first clinical application within the context of a bone marrow-purging programme in the treatment for Ph-positive leukaemia.

Other specific translocations have also been identified which offer potential targets for an antisense approach. These include the t(14;18) in B-cell lymphoma, where a *bcl-2*/immunoglobulin gene fusion is formed. Also the t(15;17) in acute promyelocytic leukaemia where the genes for the retinoic acid receptor α and the zinc finger protein PML become fused.

Many genes involved in cancer exert their effect by overexpression, or temporally inappropriate expression, while their gene products are structurally normal. These could all be considered as potential targets. Examples include c-*fos*, c-*myc*, N-*myc*, c-*erb*B-2 and the nucleolar antigen p120. Other genes of this category would include those capable of participating in autocrine and paracrine signalling loops. Proteins such as the fibroblast growth factors, the haemopoietic colony-stimulating factors, interleukins and of course their respective receptors fall into this class. Antisense approaches have been used to down-modulate the expression of several genes *in vitro*, including some of the above examples (Dolnick, 1991; Hélène & Toulmé, 1990; Stein & Cohen, 1988).

There are important caveats to the notion that a single gene product provides a clinically useful target. The cancer cell is often not the end-point of a strict linear pathway. Carcinogenesis is a dynamic process involving clonal evolution and tumor heterogeneity. For example, the acquisition of a particular activated oncogene may be an important event during tumour initiation or establishment, but may no longer be required for the survival of a fully malignant metastatic cell. The window of opportunity for intervention with an agent designed to interfere with that oncogene may have passed, possibly years previously, during the pre-clinical phase of the disease. We are not yet at a stage where we can say how great a potential problem this will be. The first goal of this technology is to identify which oncogenes are dominant and thus present themselves as useful therapeutic targets.

A further caveat to the potential application of nucleic acid-based strategies is the fact that many human tumours arise as much because of a reduction or complete loss of specific gene expression as because of overexpression. Most tumour suppressor genes are probably inappropriate candidates for strategies which seek to nullify gene expression. However, the identification of dominant negative mutations in some genes such as p53 and the recognition of genes which encode factors which reduce tumour suppressor activity (for instance MDM2 which can bind p53) may provide targets for intervention.

### What end-points can we seek with antisense therapy?

Down-regulation of a dominant oncogene, or the interruption of an autocrine or paracrine signalling loop, could be predicted to by cytostatic and not necessarily cytotoxic. This will mean that an antisense agent given exogenously will require repeated administration at appropriate intervals to ensure a continous suppression of tumour growth.

One might prefer to deliver the agent to a target whose down-regulation would produce differentiation, for example c-*myb* in macrophage differentiation. Better still one might find a target whose down-regulation would induce apoptosis and a tumour-killing effect, such as the *bcl*-2 gene. It may also be clinically useful to inhibit the metastatic potential of the tumour. The recent work of Arch *et al.* (1992) showing that the expression of a variant CD44 antigen can confer metastatic properties on non-tumorigenic cell lines, and that expression of this variant can be detected in the margins of colorectal tumours provides an attractive and useful possibility for down-modulation by an antisense approach.

A third notional target area would be what Dolnick (1991) has termed 'host-directed' targets. Examples would include down-modulation of the MDR1 gene increasing the sensitivity of the target tissue to conventional cytotoxic drugs. Alternatively, suppression of enzymes for drug activation selectively in normal tissues could allow increased doses of cytotoxics to be given for the tumour. One study has shown that antisense-mediated down-modulation of c-*raf*1 mRNA, can lead to enhanced radiation-sensitivity and reduced tumorigenicity in a radiation-resistant human squamous carcinoma cell line (Kasid *et al.*, 1989). Similarly, in ovarian carcinoma cell lines a ribozyme targeting c-*fos* mRNA caused potentially useful pleiotropic effects such as reduced expression of DNA polymerase, and increased sensitivity to cisplatin (Scanlon *et al.*, 1991).

### Types of agent currently under evaluation

Antisense and anti-gene agents currently under test are single-stranded oligodeoxynucleotides and chiefly their methylphosphonate, phosphorothioate and α-oligodeoxynucleotide analogues. The structures of these common forms are shown in Figure 1. Other analogues are undergoing development, and indeed an approach under intense investigation is the design of non-nucleotide synthetic structures which mimic nucleic acids, so called 'plastic DNA' (Uhlmann & Peyman, 1990). Novel oligodeoxynucleotide analogues in which the phosphodiester backbone is replaced with a polyamide (peptide nucleic acids, PNAs) look particularly promising (Nielsen *et al.*, 1991; Hanvey *et al.*, 1992).

A basic principle in the design of antisense molecules is to achieve a balance between conferring sufficient specificity to the target sequence and maintaining useful physio-chemical properties to ensure cell penetration and maintenance of adequate concentration for the desired biological activity. Despite the apparently minor structural differences between the various analogues, clear differences in their activities can be shown.

The unmodified phosphodiester oligodeoxynucleotides are fairly readily taken up by cells and are relatively non-toxic in cell types tested so far. They are very efficient at hybrid formation and induce RNAse H activity (see later) in heteroduplexes with RNA. The major disadvantage of the unmodified oligodeoxynucleotides is their extreme sensitivity to degradation by cellular and extracellular nucleases. Their half-life in serum is limited to a few hours, and under some conditions can be as little as 15 min.

The methylphosphonate oligodeoxynucleotides are less soluble than the phosphodiester linkage oligodeoxynucleotides and the other common analogues, but they have relatively good cell uptake characteristics with low toxicity and high stability. The major disadvantage of this class of analogue is relatively poor hybridisation efficiency and inability to induce the activity of RNase H.

The phosphorothioate oligodeoxynucleotides would seem to have properties which combine the best features of the unmodified and methylphosphonate oligodeoxynucleotides; good solubility, hybridisation efficacy, RNase H induction, and nuclease resistance. However, they appear to show sequence non-specific toxicity in some systems, and are also less well taken up by cells. Sequence-independent toxicity may be mediated by competitive inhibition of DNA polymerase α and β, and non-competitive inhibition of the γ and σ polymerase (Gao *et al.*, 1992). The inhibitory effects appear to be related to the total number of thioate linkages and not the position of the linkages within the chain. Low concentrations of phosphorothioate enhance RNase H cleavage whereas at concentrations exceeding that of the target RNA RNase H is inhibited, protecting the complementary RNA from degradation (Gao *et al.*, 1992).

The fourth major type of oligodeoxynucleotide are the α-oligodeoxynucleotides. In these, the linkage formed by the deoxyribose with the purine or pyrimidine base in normal phosphodiester linkages is in the unnatural α position. Such α-oligodeoxynucleotides form parallel rather than anti-

**Figure 1** Structure of oligodeoxynucleotides and common analogues. Oligodeoxynucleotide structure showing some of the common analogues achieved by substitution at the internucleotide phosphate. B = purine or pyrimidine base. I, 'Natural' phosphodiester linkage; II, Methylphosphonate linkage; III, Phosphorothioate linkage; IV, Linkage of the α-anomer nucleotide; V, R = -O-CH3: Methylphosphotriester, R = -O-CH2-CH3: Ethylphosphotriester, R = -NH-CH3: Alkylphosphoramidate, R = Many other substituents possible.

parallel duplexes with complementary DNA or RNA strands. These analogues show good solubility and stability. They are able to hybridise at least as efficiently as the phosphorothioates, but they do not induce RNAse H.

Some workers have exploited 'conjugated' oligodeoxynucleotides, i.e. those with particular reactive groups added to either the 5′ or 3′ end. The addition of such moieties to normal phosphodiester oligodeoxynucleotides to some extent stabilises them against nuclease attack. Such appendages may also improve cellular uptake if they are hydrophobic. Fluorescent groups provide markers for uptake and localisation, or can stabilise hybrid formation by intercalation with a double-stranded target sequence. Some of the conjugates can be catalytic centres, promoting strand cleavage at a specific target. Although such functionalisation is usually at the expense of Tm or sequence specific recognition, such approaches have seen particular application in anti-gene strategies.

An important factor to be considered in the use of some oligodeoxynucleotide analogues is stereoisomerism. This problem is raised once substitution at the internucleotide phosphate is achieved, and is a particular problem where relatively large substituents, such as the methyl group in the methylphosphonates, are introduced. Modification at $n$ phosphodiester linkages will given given $2^n$ iso-forms. Thus for a 15-mer (14 internucleotide linkages) there will be 32,568 stereoisomers. Not all isomers can bind their complementary target equally well. Some workers have used affinity purification to separate low from high affinity stereoisoforms of 9-mer methylphosphonates (Tidd *et al.*, 1988). Hybridisation experiments showed the $T_m$ of the high and low affinity populations to differ by 7.6%.

Peptide nucleic acids are able to invade duplex DNA, causing displacement of one strand and the formation of a very stable 'D-loop' which blocks RNA polymerase II-mediated transcription. However, despite the advantage of high stability in serum and within the cell, there are significant problems to be overcome. These agents can bind DNA in either orientation (which increases the potential toxicity) and binding is relatively poor at physiological salt concentrations.

### Ribozymes

A further class of antisense agent which merits consideration comprises the catalytic RNAs or ribozymes. Their structure and mode of action are entirely different to the systems we have described, but the outcome of their application and the kinds of target to which they might be directed are similar. Ribozymes are small oligo-ribo-nucleotides which have a specific base sequence with natural self-splicing activity. This activity can be directed against virtually any RNA target by the inclusion of an antisense region into the ribozyme, but at the moment there is still a need for a consensus GUC sequence in the target at the desired cleavage site. This, for instance, precludes being able to design ribozymes which can target many of the common *ras* gene mutations, but this situation may not always hold true. A PCR-based '*in vitro* evolution' technique has been recently shown capable of producing a population of ribozymes with a 100-fold enhancement in target cleavage activity compared to the starting molecule (Beaudry & Joyce, 1992). It may be possible to use such an approach to alter target specificity to suit any desired sequence. An example of a typical 'hammerhead' ribozyme is shown in Figure 2.

A major disadvantage of ribozymes at present is that, being ribonucleic acids, they are particularly sensitive to nuclease degradation. Ribozymes are not generally being considered as agents which may be exogenously administered. Whilst it should be possible to develop nuclease-resistant ribonucleotide analogues, present strategies employing ribozymes achieve their delivery by genetic means, in the form of mini-gene constructs (Cameron & Jennings, 1989). In one study (Cotten & Birnstiel, 1989), the ribozyme RNA has been expressed in stablised form as a segment within a modified tRNA gene.

### Anti-gene oligodeoxynucleotides, triple-helix agents

Oligodeoxynucleotides designed as anti-gene agents (those which target double-stranded DNA) are now usually referred to as 'triple-helix' formers or agents. It is usual for the oligodeoxynucleotide to be conjugated with a reactive group to provide a centre for specific strand cleavage, and also to stabilise and enhance hybridisation formation. Triple-helix formation is presently limited to homopurine-homopyrimidine sequences within the target DNA, and many important regulatory elements do not contain such sites. Moreover, third-strand binding is sensitive to physiological pH, requires protonated C residues (a pH effect) and is rapidly destabilised by transcription and DNA replication. The triple helix may also be susceptible to DNA repair processes within the cell. However, recent results have shown some relaxation in the target sequence requirements for third-strand binding. Some mixed sequence sites may be recognised by oligodeoxynucleotides in which G residues of the incoming third strand form stable base triplets with T-A base pairs on the target DNA. This has been shown to occur where the T residues interrupt a homopurine site (Griffin & Dervan, 1989). Another approach using pyrimidine oligodeoxynucleotides linked at their 3′ termini has shown that adjacent homopurine sites on opposite DNA strands can be successfully targeted (Horne & Duervan, 1990). The linked oligodeoxy-

**Figure 2** Structure of an H-*ras* ribozyme. The structure of a ribozyme targeting H-*ras* mutated at codon 12. The complementary H-*ras* RNA (1688–1714) is shown with the GUC cleavage site found in a mutated but not normal H-*ras* allele (Adapted from Kashani-Sabet *et al.*, 1992).

nucleotide is seemingly able to bind in the major groove of both purine strands in the target.

Passively acting (i.e. not containing a reactive group) triple-helix forming agents have been used to bind regions of DNA which are not simple homopurine-homopyrimidine tracts. Cooney *et al.* (1988) have shown inhibition of c-*myc* transcription by the presence of an upstream triple-helix in an *in vitro* system. Recently, Mergny *et al.* (1992) have shown sequence-specific inhibition of transcription initiation by triple-helix agents. This *in vitro* reaction could be greatly enhanced by the addition of a benzo[e]pyridoindol derivative. The binding of this agent to the triple-helix enhanced the stability of the triplex by as much as a 20°C increase in $T_m$. Interestingly, some of the benzo[e]pyridoindol derivatives have themselves shown promise as new anti-tumour agents (Nguyen *et al.*, 1992), where they may act by interaction with DNA topoisomerase II/DNA complexes. It remains to be tested whether or not these agents used in conjunction with triple-helix forming oligodeoxynucleotides can provide the basis for specific inhibition of transcription *in vivo*.

### Mechanisms of action

Several potential mechanisms by which antisense agents can lead to the downregulation of a particular gene can be identified. The possible mechanisms have been divided into three classes: passive, reactive and activating processes (Rothenburg *et al.*, 1989).

A passive process would be one in which the oligodeoxynucleotide exerts its effect by simple blocking of function through steric hindrance. Such a mechanism could affect many aspects of the functioning of an mRNA molecule. For example, interaction with ribosomes or splicosomes or even the transport processes from the nucleus to the cytoplasm.

Reactive processes would be where the oligodeoxynucleotide can directly cross-link with the target sequence to irreversibly block its action or, more favourably, those where the target sequence is bound and cleaved. While the former reaction will be stoichiometric, the latter might act catalytically thereby reducing the amount of compound required.

An activating process would occur when the heteroduplex formed between the target and the antisense agent, causes the activation of endogenous enzymes such as RNAse H to cleave the RNA or perhaps (in)activation of other stability modifiers.

### Sequence specificity and the significance of RNase H

RNase H digests the RNA component of a RNA–DNA heteroduplex. Thus in the context of an antisense oligodeoxynucleotide bound to its target mRNA, the RNA will become inactivated regardless of the position on which the antisense agent binds the RNA. As this mechanism is catalytic, it has the advantage that one antisense molecule can inactivate many target molecules. It is probable that RNase H-mediated cleavage is the major mechanism by which antisense effects targeted against the translated region of mRNA are brought about, although methylphosphonate and 2′-O-allyl modified phosphodiester oligodeoxynucleotides can produce translation arrest *in vitro* via an RNase H-independent mechanism.

Inhibitory activity of antisense molecules which target the 5′ untranslated region of the mRNA are also probably influenced by RNAse H, to augment the steric effects of blocking ribosome assembly or passage.

RNase H is an enzyme involved in DNA replication and as such it is likely to be widely and constitutively expressed. At the present time, its cellular and tissue distribution (particularly in tumour tissue) is not well known and may limit therapeutic approaches based on this mechanism.

A major potential source of toxic side effects with antisense agents will result from fortuitous hybridisation with non-target RNAs. It has been estimated that the oligodeoxynucleotide length required to specify a unique sequence of human mRNA is 11 nucleotides if the target contains only G and C residues, and 15 nucleotides for targets containing only A and T (Hélène & Toulme, 1989). Hence the average shortest unique sequence in the mRNA pool is 13 bases. There is evidence that a single internal mismatch within a 13-mer will not prevent degradation, indeed Woolfe *et al.* (1992) have shown that a complementary sequence of ten consecutive bases is sufficient to produce antisense degradation in a *Xenopus* oocyte system. Any given 13-mer will contain four different internal 10-mers and so could recognise any many as 76 different complementary sites. Longer oligomers will contain more internal 10-mers, and since flanking sequences do not prevent antisense effects (Woolfe *et*

*al.*, 1992), increasing the length of an oligomer is likely to increase rather than decrease the number of RNAs that will suffer non-specific degradation. However, it should be pointed out that experiments with *Xenopus* oocytes are carried out at temperatures 15–20° lower than those in mammalian cells. Hybridisation conditions will be much more restrictive in mammalian cells and it is unlikely that 10 base pairs out of 13 will be sufficient to induce additional cleavage. Despite this, such arguments underscore the importance of chain length in the design of oligodeoxynucleotides.

Problems of specificity which apply to oligodeoxynucleotides and ribozyme-based systems extend beyond statistical estimation of target sequence abundance. Kinetic considerations such as hybrid stability, rates of formation and dissociation and even location within particular cellular compartments will influence the ability of RNase H to degrade non-target RNAs. Other influential factors which are difficult to predict include rates of replacement of RNA pools and the accessibility (secondary structure) of the cross-hybridising sequence. A useful discusson of this area has been given by Herschlag (1991).

These inappropriate effects due to partial sequence hybrids are a potential problem for all oligodeoxynucleotides capable of directing RNase H. One way around this problem (Giles & Tidd, 1992), is the use of chimeric oligodeoxynucleotides, containing segments of sequence made up of methylphosphonate linkages which do not direct RNase H, surrounding an 'active window' of phosphodiester-linked nucleotides capable of directing cleavage.

### What effects have been seen with antisence agents?

In general, three types of experimental system have been exploited in assessing antisense effects. Cell-free translation systems, *Xenopus* oocytes and cells grown in tissue culture.

In experiments with cell-free translation systems, the ability of different antisense agents to inhibit the *in vitro* translation of particular mRNAs can be readily assessed. Such experiments have indicated that the best target sites in the mRNA are at the 5′ end, around the initiator AUG codon and the assembly sites for the ribosome complex. The translation initiation factors assemble in a process which probably also requires recognition of the 5′ methyl-G cap of mature mRNA molecules. Antisense molecules complementary to this region of the message are more active if their sequence extends to include a short dC tail, presumably capable of hybridising with the 5′ cap (Goodchild *et al.*, 1988).

Successful and specific inhibition of oncogene expression and tumour cell growth has been reported with antisense oligodeoxynucleotides against the message for c-*myc* (Wickstrom *et al.*, 1988; Holt *et al.*, 1988), c-*myb* (Gewirtz & Calabretta, 1988), PCNA cyclin (Jaskulski *et al.*, 1988), retinoic acid receptor α (Cope & Wille, 1989) and c-*fos* using a ribozyme approach (Scanlon *et al.*, 1991). The p120 nucleolar antigen expressed in most human malignant tumours, and of some prognostic significance in breast cancer, has been inhibited to biological effect by a genetic antisense approach (Perlaky *et al.*, 1992).

Examples of antisense inhibition of autocrine stimulation loops have also been produced. Agents targeted to CSF1 and its receptor c-*fms* can inhibit cell proliferation (Birchenall-Roberts *et al.*, 1990; Wu *et al.*, 1990). Similarly, antisense inhibition has been reported for interleukins 2, 4 and 6 (Harel-Bellan *et al.*, 1988; Schwab *et al.*, 1991), EGF receptor (Moroni *et al.*, 1992) and basic fibroblast growth factor (Murphy *et al.*, 1992).

It may be significant that in most of these cases the half-life of the mRNA is less than an hour and that complete loss of mRNA translation was not achieved, nor seemingly required, for a biological effect to be scored.

In the case of the *ras* family of proto-oncogenes, where the turnover of the protein product and its mRNA is comparatively slow, biological effects have also been noted. All three *ras* genes have been targeted by antisense approaches. N-*ras* has been down-modulated in haemopoietic cells *in vitro* (Skorski *et al.*, 1992). Ki-*ras* has been targeted using a genetic antisense approach (Mukhopadhyay *et al.*, 1991) in which a human lung cancer cell line containing a homozygous Ki-*ras* mutation was engineered to express a Ki-*ras* antisense cDNA. Cells remained viable, but showed a 3-fold reduction in growth rate and concomitant reduction in tumorigenicity.

The Ha-*ras* gene has seen the most experimental attention, targeted by various antisense oligodeoxynucleotide analogues (Brown *et al.*, 1989; Daaka & Wickstrom, 1990; Chang *et al.*, 1991), and in some studies by ribozymes (Kashani-Sabet *et al.*, 1992; Koizumi *et al.*, 1992). A remarkably high degree of specificity was achieved for mutant-specific antisense oligodeoxynucleotides using a cell-free system targeting Ha-*ras* (Saison-Behmoaras *et al.*, 1991). The 9-mers used in this study were linked to an intercalating agent (5′-acridine) and/or a hydrophobic tail (3′-dodecanol). Addition of such appendages increased the binding affinity of these short oligodeoxynucleotides resulting in RNase H-dependent specific inhibition of the mutant p21 mRNA translation, whilst the normal message was only marginally affected. Growth inhibition was achieved when similar agents were added to T24 bladder carcinoma cells, under conditions where cells containing wild-type Ha-*ras* were not affected. Similar results were produced using other analogues and cell lines (Brown *et al.*, 1989; Daake & Wickstrom, 1990; Chang *et al.*, 1991). For all of these studies, including the genetic antisense inhibition of Ki-*ras* (Mukhopadhyay *et al.*, 1991), cell lines were used which were expressing high levels of the mutant form of protein, in the absence of the competing normal mRNA. The next stage will be to show that similar effects can be achieved in cells expressing more physiological levels and ratios of mutant to wild-type p21.

### Delivery systems for antisense agents

It is obvious that the therapeutic applications of antisense nucleic acids are at a very early level in development. Manufacturing issues such as cost factors and which analogue(s) warrant scale-up production of synthesis are a long way off. Despite this, some consideration of the pharmacological aspects of antisense therapeutic strategies is warranted.

Exogenous infusion is the most direct method of delivery for any drug. Oligodeoxynucleotides are apparently cleared from the circulation in mice fairly rapidly and distributed to most tissues (Agrawal *et al.*, 1992; Zon, 1989), and preliminary toxicological studies have shown that a dose of 100 mg $kg^{-1}$ body weight of phosphorothioate oligodeoxynucleotide for 14 days is non-toxic in mice. In most tissues the oligodeoxynucleotide was quite stable, but in liver and kidney there was apparent extension of the synthetic molecule. Much of the administered dose is excreted in the urine over 2 or 3 days (more rapidly for oligodeoxynucleotides with phosphodiester linkages) and very little in the faeces.

Oligodeoxynucleotides are undoubtedly able to enter living cells. However there is evidence that much of the material remains sequestered in the extracellular environment of an endosome. It is probable that a combination of mechanisms lead to uptake, including endocytosis, and receptor-mediated internalisation. The presence of hydrophobic moieties linked to the 5′ or 3′ end of an oligodeoxynucleotide can greatly influence the rate of uptake into the cell and its intracellular distribution. One recent study (Boutorine *et al.*, 1992) showed that the addition of a porphyrin to the terminal phosphate of a 17-mer caused a 6-fold increase in uptake into T24 cells, while a 30–100 fold increase was achieved by addition of cholesterol to the oligodeoxynucleotide. On the down-side however, addition of cholesterol onto the oligodeoxynucleotide will dramatically reduce the Tm of the molecule.

The idea of including a terminal modification to enhance uptake has been taken a stage further by the report of successful delivery of antisense c-*myb* oligodeoxynucleotides to HL60 leukaemia cells, via receptor-mediated uptake. In this study (Citro *et al.*, 1992) the oligodeoxynucleotide was linked to a transferrin/polylysine complex. The interaction of this conjugate with the transferrin receptor was demonstrated by competitive inhibition with a fluoresceinated anti-transferrin receptor monoclonal antibody. Cells treated with the conjugated antisense c-*myb* agent showed loss of proliferation and viability greater than that exhibied by cells which had been treated with unconjugated antisense oligodeoxynucleotides. Such a study indicates that other ligand-receptor combinations might also be useful in the selective delivery of antisense oligodeoxynucleotides.

In studies of cellular uptake, it is important to show that the internalised oligodeoxynucleotide is intact. As has been pointed out by Tidd (1990), a particular difficulty when using oligodeoxynucleotides which have been $^{32}P$ end-labelled is the removal of label by phosphomonoesterase and incorporation of the radioactivity into cellular pools. Difficulties can also arise when using fluorescent labels as reporter groups. Tidd (1990) has suggested that certain results (Stein *et al.*, 1988; Loke *et al.*, 1988; 1989) using flow cytometry to follow a 5′ acridine label can be explained on the basis of degradative mechanisms preceding uptake. Labelling of phosphorothioate oligodeoxynucleotides with $^{35}S$ has proved effective *in vitro* (Agrawal *et al.*, 1992).

The subsequent distribution of the oligodeoxynucleotide within the cell may lead to loss of activity, for example if the molecule is locked inside endosomal vesicles. Some studies have been carried out on the effectiveness of using lipofusion as a method for introducing oligodeoxynucleotides into the cytoplasm. Not only does this method allow for the delivery of larger and/or modified oligodeoxynucleotides, it also protects the agent from extracellular degradation. However, liposome contents are primarily delivered to endosome/lysosome compartments where their contents are subjected to enzymatic degradation. Use of pH-sensitivie liposomes which release their contents following acid pH-induced fusion may circumvent this problem. Such vesicles can be made to provide receptor-mediated endocytosis and effective cytoplasmic release (Conner & Huang, 1986). Loke *et al.* (1988) have used liposome fusion to deliver antisense phosphorothioate oligodeoxynucleotides to haemopoietic cells growing in culture. In these experiments c-*myc* mRNA was targeted resulting in reduction in c-*myc* protein level, a reduced rate of DNA synthesis and growth inhibition.

There is an increasing optimism that antisense molecules could be delivered as genetic antisense agents, carried and expressed by engineered retroviruses, or delivered by transfection *in vitro* into cells which are re-infused into the patient. To date 15 clinical trials employing retroviral delivery systems have been approved in the USA (Miller, 1992). Such vectors are highly efficient at delivering genes into dividing cells. Gutierrez *et al.* (1992) have outlined a number of conditional promoters which might be included in a construct to provide tissue-specific expression of any gene or anti-gene vectored into a cell which is expressing the appropriate transcription machinery. Examples of such promoters include tyrosinase for melanoma, prostate-specific antigen for prostatic disease, polymorphic epithelial mucin for breast and pancreatic cancers. Much work remains to be done before such systems become suitable for clinical use.

### Concerns and conclusions

There are important reservations to be addressed before we can say that antisense reagents really offer useful new therapeutic opportunities. If we examine some of the above mentioned effects, aside from establishing the principle that antisense strategies can work in certain highly controlled (and carefully chosen) model systems, is there really any prospect that a new therapeutic principle has been established? Clearly biological responses in terms of 'reduced proliferation', or differentiation have undoubtedly been shown. However, with a few exceptions, virtually no studies provide direct evidence that sequence-specific recognition by the oligodeoxynucleotide was responsible for the biological phenotype(s) observed. Also, what of the magnitude of the responses, are they significantly greater than those achievable with conventional approaches? There will be little advantage in gaining increased specificity with the new agents at the expense of poor efficacy. Questions of cost effectiveness cannot be addressed until scaled-up production of the appropriate agents has been achieved. We have little idea if these agents are likely to be immunogenic or toxic in long term administration. We would predict that the types of synthetic oligodeoxynucleotide in experimental use now are unlikely to be those which ultimately find clinical use. Other nucleic acid analogues, 'plastic-DNA', PNA and ribo-oligomers, all have potential advantages which need to be explored *in vitro* and *in vivo*. The genetic delivery of antisense technology by retroviruses and possibly minichromosomes or other systems looks set to make advances in the future.

We have paid some attention to the types of target that may be useful in ablating cancer cells. High prevalence of a given molecular lesion in a particular tumour may suggest it as a tempting target, but its ablation may be sufficient to destroy the cell. Much more basic information about both the disease and antisense technology itself is still required before we can offer new therapeutic agents for the treatment of cancer, but the signs are hopeful.

### References

AGRAWAL, S. TEMSAMANI, J. & TANG, J.Y. (1992). Pharmacokinetics, biodistribution and stability of oligodeoxynucleotide phosphorothioates in mice. *Proc. Natl Acad Sci. USA*, **88**, 7595–7599.

ARCH, R., WIRTH, K., HOFMANN, M., PONTA, H., MATZKU, S., HERRLICH, P. & ZOLLER, M. (1992). Participation in normal immune responses of a metastasis-inducing splice variant of CD44. *Science*, **257**, 682–685.

BEAUDRY, A.A. & JOYCE, G.F. (1992). Directed evolution of an RNA enzyme. *Science*, **257**, 635–641.

BIRCHENALL-ROBERTS, M., FERRER, C., FERRIS, D., FALK, L.A., KASPER, J., WHITE, G. & RUSCETT, F.W. (1990). Inhibition of murine monocyte proliferation by a colony stimulating factor-1 antisense oligodeoxynucleotide. Evidence for autocrine regulation. *J. Immunol.*, **145**, 3290–3296.

BOUTORINE, A.S., BOIZIAU, C., LE DOAN, T., TOULMÉ, J.J. & HÉLÈNE, C. (1992). Effect of the terminal phosphate derivitization of β and α-oligodeoxynucleotides on their antisense activity in protein biosynthesis, stability and uptake by eukaryotic cells. *Biochemie.*, **74**, 485–489.

BROWN, D., YU, Z., MILLER, P., BLAKE, K., WEI, C., KUNG, H.F., BLACK, R.J., TS'O, P.O.P. & CHANG, E.H. (1989). Modulation of ras expression by anti-sense, nonionic deoxyoligonucleotide analogs. *Oncogene Res.*, **4**, 243–252.

CAMERON, F.H. & JENNINGS, P.A. (1989). Specific gene suppression by engineered ribozymes in monkey cells. *Proc. Natl Acad. Sci. USA*, **86**, 9139–9143.

CHANG, E.H., MILLER, P.S., CUSHMAN, C., DEVADAS, K., PIROLLO, K.F., TS'O, P.O.P. & YU, Z.P. (1991). Antisense inhibition of ras p21 expression that is sensitive to a point mutation. *Biochemistry*, **30**, 8283–8286.

CITRO, G., PERROTTI, D., CUCCO, C., D'AGNANO, I., SACCHI, A., ZUPI, G. & CALABRETTA, B. (1992). Inhibition of leukaemia cell proliferation by receptor-mediated uptake of c-*myb* antisense oligodeoxynucleotides. *Proc. Natl Acad. Sci. USA*, **89**, 7031–7035.

COHEN, J.S. (1991). Antisense oligodeoxynucleotides as antiviral agents. *Antiviral Res.*, **16**, 121–133.

COONEY, M., CZERNUSZEWICZ, G., POSTAL, E.H., FLINT, S.J. & HOGAN, M.E. (1988). Site-specific oligonucleotide binding represses transcription of the human c-*myc* gene *in vitro*. *Science*, **241**, 456-459.

COPE, F.O., & WILLE, J.J. (1989). Retinoid receptor antisense DNAs inhibit alkaline phosphatase induction and clonogenicity in malignant keratinocytes. *Proc. Natl Acad. Sci. USA*, **86**, 5590-5594.

CONNER, J. & HUANG, L. (1986). pH-sensitive immunoliposomes as an efficient carrier for antitumor drugs. *Cancer Res.*, **46**, 3431-3435.

COTTON, M. & BIRNSTIEL, M.L. (1989). Ribozyme mediated destruction of RNA *in vitro*. *EMBO J.*, **8**, 3861-3866.

DAAKA, Y. & WICKSTROM, E. (1990). Target dependence of antisense oligodeoxynucleotide inhibition of c-Ha-*ras* p21 expression and focus formation in T24-transformed NIH3T3 cells. *Oncogene Res.*, **5**, 267-275.

DOLNICK, B.J. (1991). Antisense agents in cancer research and therapeutics. *Cancer Invest.*, **9**, 185-194.

GAO, W., HAN, F., STORM, L., EGAN, W. & CHEUNG, Y. (1992). Phosphorothioate oligonucleotides are inhibitors of human DNA polymerases and RNAse H: implications for antisense technology. *Mol. Pharmacol.*, **41**, 223-229.

GEWIRTZ, A.M. & CALABRETTA, B. (1988). A c-*myb* antisense oligonucleotide inhibits normal human hematopoiesis *in vitro*. *Science*, **242**, 1303-1306.

GILES, R.V. & TIDD, D.M. (1992). Increased specificity for antisense oligodeoxynucleotide targeting of RNA cleavage by RNAse H using chimeric methylphosphonodiester/phosphodiester structures. *Nucleic Acids Res.*, **20**, 763-770.

GOODCHILD, J., CARROLL III, E.C. & GREENBURG, J.R. (1988). Inhibition of rabbit $\beta$-globin synthesis by complementary oligonucleotides: identification of mRNA sites sensitive to inhibition. *Arch. Biochem. Biophys.*, **263**, 401-409.

GRIFFIN, L.C. & DERVAN, P.B. (1989). Recognition of thymidine: adenine base pairs by guanine in a pyrimidine triple helix motif. *Science*, **245**, 967-971.

GRUNICKE, H.H. (1991). The cell membrane as a target for cancer chemotherapy. *Eur. J. Cancer*, **27**, 281-284.

GULLICK, W.J. (1990). Inhibitors of growth factor receptors. In *Genes and Cancer*, Carney, D. & Sikora, K. (eds), pp. 263-274. Wiley: Chichester, UK.

GUTIERREZ, A.A., LEMOINE, N.R. & SIKORA, K. (1992). Gene therapy for cancer. *Lancet*, **339**, 715-721.

HANVEY, J.C., PEFFER, N.J., BISI, J.E., THOMSON, S.A., CADILLA, R., JOSEY, J.A., RICCA, D.J., HASSMAN, C.F., BONHAM, M.A., AU, K.G., CARTER, S.G., BRUCKENSTEIN, D.A., BOYD, A.L., NOBEL, S.A. & BABISS, L.E. (1992). Antisense and antigene properties of peptide nucleic acids. *Science*, **258**, 1481-1485.

HAREL-BELLAN, A., DURUM, S., MUEGGE, K., ABBAS, A. & FARRAR, W.L. (1988). Specific inhibition of lymphokine biosynthesis and autocrine growth using antisense oligonucleotides in Th 1 and Th2 helper T cell clones. *J. Exp. Med.*, **168**, 2309-2318.

HAREL-BELLAN, A., BRINI, A., FERRIS, D.F., ROBIN, P. & FARRAR, W.L. (1989). In situ detection of a heat-shock regulatory element binding protein using a soluble short synthetic enhancer sequence. *Nucleic Acids Res.*, **17**, 4077-4087.

HÉLÈNE, C. & TOULMÉ, J.J. (1989). Control of gene expression by oligodeoxynucleotide covalently linked to intercalating agents and nucleic acid-cleaving reagents. In *Oligodeoxynucleotides Antisense Inhibitors of Gene Expression*, Cohen, J.A. (ed.), pp. 137-172. Macmillan Press.

HÉLÈNE, C. & TOULMÉ, J.J. (1990). Specific regulation of gene expression by antisense, sense and antigene nucleic acids. *Biochim. Biophys. Acta.*, **1049**, 99-125.

HERSCHLAG, D. (1991). Implications of ribozyme kinetics for targeting the cleavage of specific RNA molecules *in vivo*: more isn't always better. *Proc. Natl Acad. Sci. USA*, **88**, 6921-6925.

HOLT, J.T., REDNER, R.L. & NEINHUIS, A.W. (1988). An oligomer complementary to c-*myc* mRNA inhibits proliferation of HL60 promyelocytic cells and induces differentiation. *Mol. Cell. Biol.*, **8**, 963-973.

HORNE, D.A. & DERVAN, P.B. (1990). Recognition of mixed sequence duplex DNA by alternate-strand triple-helix formation. *J. Am. Chem. Soc.*, **112**, 2435-2437.22.

JASKULSKI, D., DEREIL, J.K., MERCER, W.E. CALABRETTA, B. & BASERGA, R. (1988). Inhibition of cellular proliferation by antisense oligodeoxynucleotides to PCNA cyclin. *Science*, **240**, 1544-1546.

KASHANI-SABET, M., FUNATO, T., TONE, T., JIAO, L., WANG, W., YOSHIDA, E., KASHFINN, B.I., SHITARA, T., WU, A.M., MORENO, J.G., TRAWEEK, S.T., AHLERING, T.E. & SCANLON, K.J. (1992). Reversal of the malignant phenotype by an anti-*ras* ribozyme. *Antisense Res. Development*, **2**, 3-15.

KASID, U., PFEIFER, A., BRENNAN, T., BECKETT, M., WEICHSELBAUM, R.R., DRITSCHILO, A. & MARK, G.E. (1989). Effect of antisense c-*raf1* on tumorigenicity and radiation sensitivity of human squamous carcinoma. *Science*, **242**, 1354-1356.

KOIZUMI, M., KAMIYA, H. & OHTSUKA, E. (1992). Ribozymes designed to inhibit transformation of NIH3T3 cells by the activated c-Ha-*ras* gene. *Gene*, **117**, 179-184.

LEMOINE, N.R. (1992). Mutant oncogenes: targets for therapy? *Cancer Topics*, **8**, 11

LOKE, S.L., STEIN, C., ZHANG, X., AVIGAN, M., COHEN, J. & NECKERS, L.M. (1988). Delivery of c-*myc* antisense phosphorotioate oligodeoxynucleotides to haematopoietic cells in culture by liposome fusion: specific reduction in c-*myc* protein expression correlates with inhibition of cell growth and DNA synthesis. *Current Topics in Microbiol. & Immunol.*, **141**, 282-289.

LOKE, S.H., STEIN, C., ZHANG, X.H., MORI, K., NAKANISHI, M., SUBASHINGHE, C., COHEN, J.S. & NECKERS, J.M. (1989). Characterisation of oligonucleotide transport into living cells. *Proc. Natl Acad. Sci. USA*, **86**, 3474-3478.

MERGNY, J.L., DUVAL-VALENTIN, G., NGUYEN, C.H., PERROUAULT, L., FAUCON, B., ROUGEE, M., MONTENAY-GARESTIER, T., BISAGNI, E. & HELENE, C. (1992). Triple helix specific ligands. *Science*, **256**, 1681-1684.

MILLER, D. (1992). Human gene therapy comes of age. *Nature*, **357**, 455-460.

MORONI, M.C., WILLINGHAM, M.C. & BEGUINOT, L. (1992). EGF-R antisense RNA blocks expression of the epidermal growth factor receptor and suppresses the transforming phenotype of a human carcinoma cell line. *J. Biol. Chem.*, **267**, 2714-2722.

MUKHOPADHYAY, T., TAINSKI, M., CAVENDER, A.C. & ROTH, J. (1991). Specific inhibition of K-*ras* expression and tumourigenicity on lung cancer cells by antisense RNA. *Cancer Res.*, **51**, 1744-1748.

MURPHY, P.R., SATO, Y. & KNEE, R.S. (1992). Phosphorothioate antisense oligonucleotides against basic fibroblast growth factor inhibit anchorage-dependent and anchorage-independent growth of a malignant glioblastoma cell line. *Mol. Endocrinol.*, **6**, 877-884.

NIELSEN, P.E., EGHOLM, M., BERG, R.H. & BUCHARDT, O. (1991). Sequence-selective recognition of DNA by strand displacement with a thymidine-substituted polyamide. *Science*, **254**, 1497-1500.

NGUYEN, C.H., LAVELLE, F., RIOU, J.F., BISSERY, M.C., HUEL, C. & BISAGNI, E. (1992). Further SAR in the new antitumor 1-amino-substituted gamma-carbolines and 5H-benzo[e]pyrido[4,3-b]indoles series. *Anticancer Drug Design*, **7**, 235-251.

PERLAKY, L., VALDEZ, B.C., BUSCH, R.K., LARSON, R.G., JHIANG, S.M., ZHANG, W.W., BRITTAIN, M. & BUSCH, H. (1992). Increased growth of NIH/3T3 cells by transfection with human p120 complementary DNA and inhibition by a p120 antisense construct. *Cancer Res.*, **52**, 428-436.

ROTHENBURG, M., JOHNSON, G., LAUGHLIN, C., GREEN, I., CRADOCK, J., SARVER, N. & COHEN, J.S. (1989). Oligonucleotides as antisense inhibitors of gene expression: therapeutic implications. *J. Natl Cancer Inst.*, **81**, 1539-1544.

SAISON-BEHMOARAS, T., TOCQUE, B., REY, I., CHASSIGNOL, M., THUONG, N.T. & HÉLÈNE, C. (1991). Short modified antisense oligonucleotides directed against Ha-*ras* point mutation induced selective cleavage of the mRNA and inhibit T24 cells proliferation. *EMBO J.*, **10**, 1111-1118.

SCANLON, K.J., JIAO, L., FUNATO, T., WANG, W., TONE, T., ROSSI, J.J. & KASHANI-SABET, M. (1991). Ribozyme-mediated cleavage of c-*fos* mRNA reduces gene expression of DNA synthesis enzymes and metallothionein. *Proc. Natl Acad. Sci. USA*, **88**, 10591-10595.

SCHWAB, G., SIEGALL, C.B., AARDEN, L.A., NECKERS, L. & NORDAN, R.P. (1991). Characterization of a interleukin-6-mediated autocrine growth loop in the human multiple myeloma cell line U266. *Blood*, **77**, 587-593.

SKORSKI, T., SZCYLIK, C., RATAJCZAK, M.Z., MALAGUARNERA, L., GEWIRTZ, A.M. & CALABRETTA, B. (1992). Growth factor-dependent inhibition of normal hematopoiesis by N-*ras* antisense oligodeoxynucleotides. *J. Exp. Med.*, **175**, 743-750.

STEIN, C.A. & COHEN, J.S. (1988). Oligodeoxynucleotides as inhibitors of gene expression: a review. *Cancer Res.*, **48**, 2959-2668.

STEIN, C.A., MORI, K., LOKE, S.L., SUBASHNGHE, C., SHINOZUKA, K., COHEN, J.S. & NECKERS, L.M. (1988). Phosphorothioate and normal oligodeoxyribonucleotides with 5′ linked acridine: characterisation and preliminary kinetics of cellular uptake. *Gene*, **72**, 333–341.

SZCZYLIK, C., SKORSKI, T., NICOLAIDES, N.C., MANZELLA, L., MALAGUARNERA, L.L., VENTURELLA, D., GERWIRTZ, A.M. & CALABRETTA, B. (1991). Selective inhibition of leukaemia cell proliferation by BCR-ABL antisense oligodeoxynucleotides. *Science*, **253**, 562–565.

TIDD, D.M., HAWLEY, P., WARENIUS, H.M. & GIBSON, I. (1988). Evaluation of N-*ras* oncogene anti-sense, sense and nonsense sequence methylphosphorate oligonucleotide analogues. *Anti-Cancer Drug Design*, **3**, 117–127.

TIDD, D.M. (1990). A potential role for antisense oligonucleotide analogues in the development of oncogene-targeted cancer chemotherapy. *Anticancer Res.*, **10**, 1169–1182.

UHLMANN, E. & PEYMAN, A. (1990). Antisense oligonucleotides: a new therapeutic principle. *Chemical Rev.*, **4**, 543–584.

WICKSTROM, E.L., BACON, T.A., GONZALEZ, A., FREEMAN, D.L., LYMAN, G.H. & WICKSTROM, E. (1988). Human promyelocytic leukaemia HL-60 cell proliferation and c-*myc* protein expression are inhibited by antisense pentadecadeoxynucleotide targeted against c-*myc* mRNA. *Proc. Natl Acad. Sci. USA*, **85**, 1028–1032.

WOOLFE, T.D., MELTON, D.A. & JENNINGS, C.G.B. (1992). Specificity of antisense oligodeoxynucleotides *in vivo*. *Proc. Natl Acad. Sci. USA*, **89**, 7305–7309.

WU, J., ZHU, J.Q., HAN, K.K. & ZHU, D.X. (1990). The role of the c-*fms* oncogene in the regulation of HL-60 cell differentiation. *Oncogene*, **5**, 873–877.

ZON, G. (1989). Pharmacological considerations. In *Oligodeoxynucleotides Antisense Inhibitiors of Gene Expression*, Cohen, J.A. (ed.), pp. 233–244. Macmillan Press.